# Validation of the Advanced Practice Nursing Competency Assessment Instrument in a hospital environment

**DOI:** 10.1590/0034-7167-2022-0705

**Published:** 2023-11-10

**Authors:** Júlia Altafini, Flávia Carvalho Pena Dias, Thelen Daiana Mendonça Ferreira, Pedro Sastre-Fullana, Thaís Moreira São-João, Renata Cristina Gasparino

**Affiliations:** I Universidade Estadual de Campinas. Campinas, São Paulo, Brazil; II Hospital Universitario Son Espases. Palma de Mallorca, Illes Balears, Spain; III University of Rhode Island. Kingston, Estados, United States of America

**Keywords:** Validation Study, Advanced Practice Nursing, Professional Competence, Nurses, Hospitals, Estudio de Validación, Enfermería de Práctica Avanzada, Competencia Profesional, Enfermeras y Enfermeros, Hospitales, Estudo de Validação, Prática Avançada de Enfermagem, Competência Profissional, Enfermeiras e Enfermeiros, Hospitais

## Abstract

**Objectives:**

to evaluate the measurement properties of the Advanced Practice Nursing Competency Assessment Instrument – Brazilian version, in the hospital environment.

**Methods:**

a methodological study conducted in a hospital with 238 nurses. Three instruments collect the data: sample characterization form, Brazilian version of the Advanced Practice Nursing Competency Assessment Instrument, and the category “therapeutic interventions” of the nurse competence scale. Construct validity was verified by confirmatory factor analysis and Spearman’s correlation coefficient, and reliability by Cronbach’s Alpha and composite reliability.

**Results:**

in the factor analysis, the model converged to a satisfactory result. The study found acceptable evidence of reliability (Cronbach’s Alpha, 0.76-0.87; and composite reliability, 0.85-0.90).

**Conclusions:**

the instrument demonstrated evidence of construct validity and internal consistency and can be used in practice

## RESUMO

**Objetivos:**

avaliar as propriedades de medida do Instrumento para Avaliação de Competências do Enfermeiro de Prática Avançada – versão brasileira, no ambiente hospitalar.

**Métodos:**

estudo metodológico, realizado em um hospital, com 238 enfermeiros. Os dados foram coletados por meio de três instrumentos: ficha para caracterização da amostra; versão brasileira do Instrumento para Avaliação de Competências do Enfermeiro de Prática Avançada; e a categoria “Intervenções Terapêuticas” da Escala de Competência do Enfermeiro. A validade de construto foi verificada por meio da análise fatorial confirmatória e do coeficiente de correlação de Spearman; e a confiabilidade, por meio do alfa de Cronbach e da confiabilidade composta.

**Resultados:**

na análise fatorial, o modelo convergiu para um resultado satisfatório. Foram encontradas evidências satisfatórias de confiabilidade (alfa de Cronbach, 0,76-0,87; e confiabilidade composta, 0,85-0,90).

**Conclusões:**

o instrumento demonstrou evidências de validade de construto e consistência interna e poderá ser utilizado na prática.

## RESUMEN

**Objetivos:**

evaluar las propiedades de medida del Instrumento para Evaluación de Competencias del Enfermero de Práctica Avanzada – versión brasileña, en ambiente hospitalario.

**Métodos:**

estudio metodológico, realizado en un hospital, com 238 enfermeros. Los datos fueron recolectados mediante tres instrumentos: ficha para caracterización de la muestra; versión brasileña del Instrumento para Evaluación de Competencias del Enfermero de Práctica Avanzada; y la categoría “Intervenciones Terapéuticas” de la Escala de Competencia del Enfermero. La validez de constructo verificada por meio del análisis factorial confirmatorio y del coeficiente de correlación de Spearman; y la confiabilidad, por medio del alfa de Cronbach y de la confiabilidad compuesta.

**Resultados:**

en el análisis factorial, el modelo convergió para un resultado satisfactorio. Fueron encontradas evidencias satisfactorias de confiabilidad (alfa de Cronbach, 0,76-0,87; y confiabilidad compuesta, 0,85-0,90).

**Conclusiones:**

el instrumento demostró evidencias de validez de constructo y consistencia interna y podrá ser utilizado en la práctica.

## INTRODUCTION

The current public health landscape, especially when considering the region of Latin America and the Caribbean, reveals the detriment of access and universal health coverage to citizens due to the contrasting socio-cultural, geographical, and economic realities and gender inequalities to which they are subjected^(1)^. The aging of the population, the spread of chronic diseases, the consequences of climate change, as well as internal migration between countries also constitute obstacles to qualified and resolute care concerning the health process-disease^(1, 2)^.

Faced with increasingly complex health demands and a lack of professionals, mainly due to its poor territorial distribution^(2)^, the Advanced Nursing Practice (ANP) emerges as a strategy for access to health services, as it consists of the exercise of a nurse with at least a master’s degree, integrated into the interprofessional team, capable of making complex decisions and full of clinical skills to develop an expanded role^(2, 3)^.

The scope of the practice performed by the Advanced Practice Nurse (APN) varies according to the regional context in which it is inserted. However, in general, it includes monitoring and treatment of patients with chronic diseases, diagnosis of less complex clinical conditions, request for tests, and prescription of certain medications, according to protocols and clinical guidelines^(3, 4)^.

Brazil emerges as a country with great potential to carry out this practice, as it has a range of undergraduate and graduate courses in Nursing and pilot projects that have already been developed^(5)^. In addition, the Professional Practice Law and the National Primary Care Policy guarantee autonomy and relevance to nurses, basic assumptions for the implementation of the ANP^(6, 7)^.

More recently, in addition to Primary Care, the insertion of the APN in the hospital environment has been discussed to increase these professionals’ autonomy in decisions about managed care. After all, the functions performed by nurses at this level of health care are fragmented concerning the union between management and care^(8)^.

Thus, it is essential to recognize the importance of the full development of the potential and skills of nurses, accentuating their qualification, rethinking their roles and their professional responsibilities, not only in primary care, but also in the hospital, as a way of attesting to better care outcomes^(4, 9)^.

Therefore, the study highlights the Advanced Practice Nursing^(10, 11, 12, 13, 14, 15)^ Competency Assessment Instrument APNCAI – Brazilian version for its recognized methodological rigor and its application at different levels of healthcare to map the competencies of the APN^(14, 15)^.

Due to the importance of the APN’s work in the hospital context to ensure higher quality and safety in the care offered to patients and because of the scarcity of a validated instrument for this purpose in Brazil, the following question guided the development of this project: does the Brazilian version of the APNCAI demonstrate evidence of validity and reliability when considering the hospital context?

The availability of a tool with certified measurement properties for this level of health care will enable managers to identify the competencies of the nurses with whom they work to subsequently guide the implementation of professional development strategies, which may contribute to the achievement of better results.

## OBJECTIVES

To evaluate the measurement properties of the Advanced Practice Nursing Competency Assessment Instrument APNCAI Brazilian version, in the hospital environment.

## METHODS

### Ethical aspects

Firstly, the author of the instrument authorized its use for the conduct of this research. The Research Ethics Committee of the institution approved the project which met the ethical recommendations regarding research developed with human beings following resolution 466/2012 of the National Health Council.

### Design, period, and place of study

It is a methodological study^(16)^ that evaluated the construct validity (structural and hypothesis testing) and reliability (internal consistency) of the Advanced Practice Nursing Competency Assessment Instrument APNCAI - Brazilian version^(15)^ in the hospital setting. For the description of the research, the study adopted the criteria of the checklist Consensus-Based Standards for the selection of health Measurement Instruments (COSMIN)^(17)^. The study was conducted between October 2020 and April 2021, in an online and face-to-face format, in a hospital school in the city of Campinas, State of São Paulo (SP), Brazil, which serves patients from the Single Health System (SUS). Its mission is to provide quality assistance to its users, in addition to maintaining a commitment to teaching and research.

### Population or sample; criteria of inclusion and exclusion

The sample size calculation was based on the research objective of validating the instrument through the analysis of structural validity. For this, the internationally recommended criterion was adopted as “adequate,” which considers a minimum of a hundred participants and five respondents for each item of the instrument, equivalent to 220 professionals^(17)^. The study selected participants for convenience, and considered as inclusion criterion: being a nurse, regardless of the sector of activity. Professionals who, despite having agreed to participate, left one or more items of the instrument in blank were excluded from the study.

### Study protocol

The collection took place in a hybrid manner. For online collection, the hospital’s Nursing Department made available the nurses’ emails, to which were sent, in two rounds, the virtual invitations containing a link to access the Informed Consent Form and the instruments. In the face-to-face collection, nurses were approached in their respective sectors, guided about the purpose of the study, and invited to participate. Those who accepted signed the Informed Consent Form, received the printed instruments, and delivered them completed to the researcher on the agreed day.

The instruments used for the collection were: a personal and professional characterization form of the sample, the APNCAI – Brazilian version^(15)^, and the “therapeutic interventions” category of the Nurse Competence Scale (NCS)^(18)^

### Personal and Professional Characterization Form

The form contained personal information (age, gender, and marital status) and professional data (professional training, unit, work shift, duty, time of experience as a nurse, and in the area in which he was currently working, number of jobs) to describe the sample of participants.

### Advanced Practice Nursing Competency Assessment Instrument – Brazilian version

The APNCAI – the Brazilian version, aims to evaluate the competence of nurses according to the necessary roles and standards of the ANP through 44 items distributed in eight dimensions: Research and Practice based on evidence; Clinical and Professional Leadership; Professional Autonomy; Interprofessional Relationships and Mentoring; Care Management; Teaching and Professional Education, and Health Promotion^(14, 15)^.

The composition of the dimensions are the following: Research and Practice Based on Evidence – a total of eight items (1.1, 1.2, 1.3, 1.4, 1.5, 1.6, 1.7, and 1.8), which involve the promotion of the relationship between the research and identification of scientific evidence most relevant and clinical and care practice; Clinical and Professional Leadership – four (2.1, 2.2, 2.3, and 2.4), that demonstrate the leadership of advanced practice nurses in promoting quality health care, in addition to counseling and consulting with other professionals; Professional Autonomy – eight items (3.1, 3.2, 3.3, 3.4, 3.5, 3.6, 3.7, and 3.8) concerning the assessment of autonomy in the use of pharmacological and non-pharmacological interventions, clinical diagnosis, referral to other professionals, treatments and therapies; Interprofessional Relationships and Mentoring – six items (4.1, 4.2, 4.3, 4.4, 4.5, and 4.6), which reflect the collaboration and relationship with other health care professionals to improve direct and indirect patient care and be a clinical reference for inexperienced professionals; Management of the Quality – four items (items 5.1, 5.2, 5.3, and 5.4), measuring the skills required for the assessment and the systematic promotion of the quality, effectiveness of practices and advanced care for a whole health-disease; Care Management – six items (6.1, 6.2, 6.3, 6.4, 6.5, and 6.6), which represent the coordination of care across the different levels of care in the health system; Teaching and Professional Education – four items (7.1, 7.2, 7.3, and 7.4) that are connected to the role of the educator regarding the learning of patients and their families, other nurses, students, and health professionals; and Health Promotion – four items (8.1, 8.2, 8.3, and 8.4), which focused attention on the improvement and/or recovery of the user health^(14, 15)^.

Each item of the dimensions is evaluated by a Likert scale with five points, in which the participant answers how often they perform the described competence in their current job through the following answer options: never (1 point), almost never (2 points), sometimes (3 points), almost always (4 points) and always (5 points). Therefore, the higher the score, the higher the frequency of use of the described competence in professional activities^(14, 15)^.

### Nurse Competence Scale

The NCS evaluated the construct validity through hypothesis testing^(18)^. Through 73 items distributed in seven categories, this scale aims to assess how often nurses use specific competencies. The present study used only the category “therapeutic interventions” (α = 0.87), which has ten items (39, 40, 41, 42, 43, 44, 45, 46, 47, 48) related to flexible planning of activities by nurses, coordination of the multidisciplinary team, use of institutional protocols, installation of relevant knowledge for care, and decision-making according to specific patient situations — such items reflect, in a certain way, the dimensions of the APNCAI – Brazilian version. The response scale for each item is of the Likert type with four points, ranging from 0 (does not apply to my practice) to 3 (used very often). Thus, the higher the score, the greater the frequency with which the professional uses that competence in their professional activities^(18)^.

### Analysis of results and statistics

The collected data were entered in the Microsoft Excel for Windows® program and processed by Statistical Analysis Software® version 9.4 and SmartPLS® 3.2.1 software. Categorical variables underwent descriptive analysis, and these results were organized into absolute and relative frequency tables. For the continuous variables, the study calculated the measurements of position (mean, median, minimum, and maximum) and dispersion (standard deviation).

The scores for the dimensions of the instruments were obtained by averaging the scores of the participants’ responses. In the “therapeutic interventions” category of the NCS, the answer “does not apply to my practice” was not considered in the calculation of the average.

The study applied structural validity through confirmatory factor analysis and hypothesis testing to verify the validity of the construct. The factor analysis was conducted using structural equation models^(19)^ in which the adapted version of the APNCAI for the Brazilian culture was considered a second order variable.

When evaluating the model, the study calculated the values of the average variance extracted (AVE) for each factor, and values greater than 0.5 indicated that the model converges to a satisfactory result^(20)^. It also analyzed the cross-loads to verify if the factorial load of an item was higher in the factor in which it was initially allocated; and the discriminant validity of the model using the Fornell-Larcker criterion, which verifies if the square roots of the AVE are higher than the correlations between the dimensions^(21)^.

Regarding the hypothesis test, the study formulated the following hypothesis: the higher the score in the dimensions of the APNCAI – Brazilian version, the higher the score in the category “therapeutic interventions” of the NCS. For this analysis, the research used the Spearman’s correlation coefficient^(22)^.

Reliability was evaluated through internal consistency, and, for this, Cronbach’s Alpha coefficient and composite reliability (CR) were calculated, in which values equal to or greater than 0.7 were considered acceptable^(22)^.

## RESULTS

The sample consisted of 238 nurses, of whom 31 (13%) answered the instrument online, and 207 (87%) answered it in person. The average age was 41.3 years (SD = 8.6), with the majority being female (n = 201; 84.4%), married (n = 139; 59%), with a postgraduate degree in the specialization modality (n = 136; 57.1%), working in the care function (n = 184; 77.3%) and only one institution (n = 198; 83.5%). Most of the participants worked in adult and pediatric hospitalization units (n = 101; 42.4%) on night shift (n = 67; 28.1%). It had an average professional experience of 15.3 years (SD = 8.3) and 7.9 years (SD = 7.2) in the current area.

For the evaluation of structural validity, the research excluded 55 (23.1%) participants for having left more than 50% of the items unanswered in at least one of the dimensions of the APNCAI – Brazilian version. The study calculated the AVE, CR, and Cronbach’s alpha of each dimension with the 183 participants, as can be seen in Table 1.

**Table 1:** Average variance extracted from the dimensions of the Advanced Practice Nursing Competency Assessment Instrument – Brazilian version (N = 183), Campinas, São Paulo, Brazil, 2020-2021

APNCAI* dimensions – Brazilian version	AVE†
1 - Evidence-based Research and Practice	0.50
2 - Clinical and Professional Leadership	0.58
3 - Professional Autonomy	0.52
4 - Interprofessional Relations and Mentoring	0.52
5 - Quality Management	0.65
6 - Care Management	0.56
7 - Teaching and Vocational Education	0.67
8 - Health Promotion	0.61

* *APNCAI - Advanced Practice Nursing Competency Assessment Instrument – Brazilian version;*

† *AVE – average variance extracted.*

The factor loads of the items in their respective dimensions and cross-factor loads are presented in Table 2.

**Table 2:** Factor loadings of items in their respective constructs (highlighted) and cross-factor loadings (N = 183), Campinas, São Paulo, Brazil, 2020-2021

Item	D1^*^	D2†	D3‡	D4^§^	D5	D6^¶^	D7^**^	D8^††^
1.1	0.58	0.31	0.21	0.25	0.32	0.17	0.35	0.24
1.2	0.71	0.48	0.33	0.29	0.39	0.28	0.34	0.26
1.3	0.56	0.29	0.22	0.19	0.32	0.16	0.30	0.15
1.4	0.72	0.46	0.42	0.37	0.35	0.37	0.37	0.36
1.5	0.74	0.49	0.38	0.46	0.39	0.37	0.41	0.31
1.6	0.77	0.53	0.41	0.36	0.49	0.34	0.39	0.23
1.7	0.77	0.53	0.45	0.38	0.43	0.33	0.24	0.34
1.8	0.74	0.53	0.38	0.40	0.45	0.30	0.23	0.32
2.1	0.34	0.66	0.23	0.35	0.39	0.29	0.40	0.22
2.2	0.60	0.82	0.26	0.46	0.53	0.33	0.42	0.24
2.3	0.53	0.78	0.33	0.34	0.47	0.21	0.24	0.20
2.4	0.50	0.78	0.33	0.25	0.42	0.27	0.29	0.16
3.1	0.29	0.21	0.71	0.31	0.32	0.28	0.10	0.25
3.2	0.38	0.33	0.77	0.35	0.37	0.37	0.21	0.37
3.3	0.43	0.33	0.76	0.37	0.38	0.41	0.25	0.39
3.4	0.39	0.20	0.74	0.35	0.35	0.43	0.29	0.42
3.5	0.34	0.31	0.75	0.32	0.41	0.31	0.16	0.25
3.6	0.23	0.19	0.72	0.26	0.32	0.37	0.18	0.38
3.7	0.37	0.24	0.65	0.28	0.35	0.22	0.05	0.30
3.8	0.45	0.30	0.66	0.44	0.37	0.42	0.21	0.48
4.1	0.27	0.37	0.26	0.64	0.36	0.29	0.33	0.17
4.2	0.48	0.44	0.43	0.79	0.52	0.40	0.41	0.28
4.3	0.36	0.27	0.43	0.78	0.41	0.45	0.40	0.43
4.4	0.42	0.29	0.29	0.74	0.42	0.49	0.42	0.37
4.5	0.30	0.35	0.25	0.74	0.53	0.36	0.40	0.27
4.6	0.25	0.29	0.37	0.61	0.52	0.21	0.30	0.27
5.1	0.34	0.33	0.46	0.53	0.74	0.45	0.35	0.43
5.2	0.50	0.54	0.39	0.59	0.87	0.40	0.46	0.31
5.3	0.51	0.57	0.44	0.53	0.87	0.41	0.48	0.36
5.4	0.44	0.47	0.31	0.39	0.72	0.39	0.31	0.35
6.1	0.21	0.21	0.41	0.37	0.33	0.71	0.34	0.39
6.2	0.34	0.26	0.16	0.32	0.29	0.68	0.50	0.37
6.3	0.19	0.12	0.38	0.40	0.26	0.73	0.31	0.53
6.4	0.46	0.34	0.44	0.45	0.48	0.82	0.45	0.55
6.5	0.35	0.38	0.33	0.36	0.45	0.78	0.44	0.42
6.6	0.31	0.28	0.49	0.42	0.45	0.77	0.42	0.62
7.1	0.36	0.31	0.16	0.40	0.35	0.48	0.79	0.30
7.2	0.45	0.40	0.28	0.41	0.42	0.41	0.83	0.38
7.3	0.39	0.42	0.22	0.48	0.49	0.44	0.85	0.37
7.4	0.31	0.34	0.19	0.43	0.38	0.47	0.82	0.30
8.1	0.34	0.29	0.27	0.30	0.36	0.42	0.32	0.61
8.2	0.32	0.27	0.45	0.38	0.40	0.51	0.36	0.82
8.3	0.28	0.15	0.44	0.29	0.30	0.53	0.26	0.83
8.4	0.30	0.14	0.38	0.33	0.33	0.55	0.35	0.84

^*^ D1 – Dimension 1 (Evidence-Based Research and Practice); ^†^D2 – Dimension 2 (Clinical and Professional Leadership); ^‡^D3 – Dimension 3 (Professional Autonomy); ^§^D4 – Dimension 4 (Interprofessional Relations and Mentoring); ^||^D5 – Dimension 5 (Quality Management); ^¶^D6 – Dimension 6 (Care Management); ^**^D7 – Dimension 7 (Education and Vocational Education); ^††^D8 – Dimension 8 (Health Promotion).

The square root of the AVE and the correlations between the constructs are shown in Table 3.

**Table 3:** Discriminant validity of the factorial model, according to the Fornell-Larcker criterion, of the Advanced Practice Nursing Competency Assessment Instrument – Brazilian version (N = 183), Campinas, São Paulo, Brazil, 2020-2021

APNCAI* dimensions – Brazilian version	1	2	3	4	5	6	7	8
1- Evidence research and practice	0.70							
2- Clinical and professional leadership	0.65	0.76						
3 - Professional autonomy	0.51	0.37	0.72					
4- Interprofessional relations and mentoring	0.49	0.46	0.47	0.72				
5 - Quality Management	0.56	0.60	0.50	0.64	0.80			
6 - Care Management	0.42	0.36	0.50	0.52	0.51	0.75		
7- Teaching and Vocational Education	0.46	0.45	0.26	0.53	0.50	0.55	0.82	
8- Health Promotion	0.40	0.27	0.50	0.42	0.45	0.65	0.41	0.78

* *APNCAI – Advanced Practice Nursing Competency Assessment Instrument – Brazilian version.*

The analysis of internal consistency, evaluated by composite reliability and Cronbach’s Alpha, was shown in Table 4.

**Table 4:** Composite reliability and Cronbach’s alpha of the dimensions of the Advanced Practice Nursing Competency Assessment Instrument – Brazilian version (N = 183), Campinas, São Paulo, Brazil, 2020-2021

APNCAI* dimensions – Brazilian version	Composite reliability	Cronbach’s Alpha
1 - Evidence-based Research and Practice	0.89	0.85
2 - Clinical and Professional Leadership	0.85	0.76
3 - Professional Autonomy	0.90	0.87
4 - Interprofessional Relations and Mentoring	0.87	0.81
5 - Quality Management	0.88	0.81
6 - Care Management	0.88	0.84
7- Teaching and Vocational Education	0.89	0.84
8- Health Promotion	0.86	0.78

* *APNCAI - Advanced Practice Nursing Competency Assessment Instrument – Brazilian version.*

To evaluate the validity of the convergent construct, the study tested the correlations between each dimension of the APNCAI – Brazilian version and the category “therapeutic interventions” of the NCS (Table 5).

**Table 5:** Correlation between the dimensions of the Advanced Practice Nursing Competency Assessment Instrument – Brazilian version and the category “therapeutic interventions” of the nurse competence scale, Campinas, São Paulo, Brazil, 2020-2021

APNCAI* dimensions – Brazilian version	“Intervenções Terapêuticas” da ECE^†^
1 - Evidence-based Research and Practice	0.3744
	**< 0.0001** ^‡^
	229
2 - Clinical and Professional Leadership	0.3450
	**< 0.0001** ^‡^
	232
3 - Professional Autonomy	0.3133
	**< 0.0001** ^‡^
	229
4 - Interprofessional Relations and Mentoring	0.4111
	**< 0.0001** ^‡^
	232
5 - Quality Management	0.3633
	**< 0.0001** ^‡^
	231
6 - Care Management	0.3719
	**< 0.0001** ^‡^
	231
7- Teaching and Vocational Education	0.4108
	**< 0.0001** ^‡^
	232
8- Health Promotion	0.3355
	**< 0.0001** ^‡^
	230

^*^APNCAI – Advanced Practice Nursing Competency Assessment Instrument – Brazilian version; ^†^ NCS – Nurse Competence Scale; ^‡^p value - obtained through Spearman’s correlation coefficient.

## DISCUSSION

The evaluation of constructs using instruments validated with methodological rigor is a significant way to guarantee the safe use of tools in clinical and research practice. There is evidence in the literature that the measurement properties of an instrument can be evaluated through validity, reliability, and responsiveness; and, because they are independent and complementary, studies should adopt more than one measurement, a recommendation employed in the present study^(17, 22)^.

The validity of an instrument reveals its ability to measure what it proposes precisely. In this study, validity was assessed using construct validity: structural and hypothesis testing^(20, 22)^.

The structural construct validity was evaluated using a structural equation model considering the PLS estimation method. It is a methodology whose application is still recent in the scientific literature and presents a smaller volume of publications compared to the traditional method based on the analysis of covariances (CB-SEM). In the latter, the evaluation of the measurement model is called confirmatory factor analysis, while in the PLS method, this evaluation is called composite confirmatory analysis^(23)^.

Although they present some differences, both methods can be used to confirm a measurement model of measuring instruments that are being developed or adapted^(23)^. One of the main differences between the two methods is that, in PLS, evaluation of goodness-of-fit measures is not required as is done in the covariance-based method^(23)^. Between the two, PLS has the advantages of obtaining more accurate estimates in situations where the sample size is small, allowing the construction of more complex models and not having as an assumption that the variables included in the analysis present normal distribution^(23)^.

In the confirmatory factor analysis, the instrument items demonstrated adequacy to the dimensions insofar as all the steps conducted in the process reached the minimum values established. In the analysis of the AVE, it was possible to see that the items of the dimensions explained more than 50% of the construct in question, that is, the competence of the APN, demonstrating that the results reflect an acceptable model^(23)^.

When analyzing the cross loads and the Fornell-Larcker criterion, the independence between the constructs was verified, demonstrating that the items are accurate and reflect the concept of the dimensions in which they were allocated^(24)^.

The results obtained in the analysis of the homogeneity of the items were compared to those of the original study, and it was possible to note that the Brazilian version achieved Cronbach’s alpha values slightly lower (0.76-0.87) than the Spanish version (0.81-0.92)^(14)^, but both the Cronbach’s Alpha and the CR of each of the dimensions were higher than the recommended minimum^(21)^. These small variations may be related to the particularities of the participating sample, the conjuncture and the pandemic moment^(25)^. It is noteworthy that, in addition to Cronbach’s Alpha, CR was also used in this study to evaluate the homogeneity of the items, as authors affirm that this measure is more robust and does not underestimate the internal consistency^(16, 17)^.

At the end of these evaluations, the study showed that the Brazilian version applied in a hospital environment did not suffer any modification in its structure when compared to the original version^(14)^. That is, it kept the 44 items distributed in the exact eight dimensions same.

The hypothesis was that the higher the score in the dimensions of the APNCAI – Brazilian version, the higher the score would be in the category “therapeutic interventions” of the AVE; and the evaluation of validity through the hypothesis test showed that the data obtained were significant, demonstrating that the more advanced practice skills the nurse has, the more skills related to therapeutic interventions he performs. This AVE category includes items that highlight the importance of evidence-based updating and the nurse’s contribution to the nursing team, multidisciplinary team, and the patient^(18)^.

### Study limitations

A limitation of this study is that the number of exclusions may have impaired the sample size, considering the factor analysis. However, even so, the tool demonstrates evidence of validity and reliability to be used in the hospital environment because the sample size for construct validity by hypothesis testing and reliability analysis was larger than the internationally recommended^(17)^.

In addition, memory bias and social desirability may have interfered with the results. Social desirability occurs when some respondents provide answers that differ from their actual attitudes, values, or behaviors^(26)^, and almost half of the studies that use questionnaires shows it^(27)^. From this perspective, participants change their responses to manage the impression caused or through self-deception (to feel good about themselves), especially when researchers interact with survey participants^(26)^. In contrast, the hybrid data collection presented here has been frequently used in the current epidemiological context^(28)^, and it brings potential benefits while helping to minimize the risk of social desirability bias.

Differences between cultures are biases or barriers to the reuse of questionnaires despite a fair share of the validated instruments face problems unless they are developed and used in a very distinct and homogeneous group during a limited period^(29)^, which would have little practical application in research development. There can be cultural differences not only between countries, but also over time, generations, nations, social classes, ethnic groups, regions, industries, professions, and organizations. In addition to the well-established benefits of using questionnaires, they allow comparisons between studies — a potentially useful fact to advance in areas of study.

As the measurement properties of an instrument are not static and may vary according to the population, mode of administration, and sample size^(^16-17^)^, further studies must reevaluate these properties to confirm them in different contexts.

### Contributions to the field of Nursing

This study brings a relevant contribution to Nursing, as it will allow the start of skills mapping of the APN in Brazil. It may be valuable for strengthening the discussions and implementation of this practice in the national territory, mapping nurses’ competencies, implementing interventions that develop the autonomy of professionals for safe and interdisciplinary practice. In addition, it can help to improve the quality and safety of care offered to patients who need hospitalization in a hospital environment useful.

## CONCLUSIONS

In the sample studied, the Advanced Practice Nursing Competency Assessment Instrument APNCAI – Brazilian version demonstrated evidence of construct validity and internal consistency and can be used in practice to assist in the mapping and, in the future, the implementation of strategies for the development of advanced practice nurses in the hospital context.
